# Early severe morbidity and resource utilization in South African adults on antiretroviral therapy

**DOI:** 10.1186/1471-2334-9-205

**Published:** 2009-12-15

**Authors:** Teresa K Smith de Cherif, Jan H Schoeman, Susan Cleary, Graeme A Meintjes, Kevin Rebe, Gary Maartens

**Affiliations:** 1 Department of Medicine, Division of Infectious Diseases, University of Cape Town, Cape Town, South Africa; 2 School of Public Health and Family Medicine, Health Economics Unit, University of Cape Town, Cape Town, South Africa; 3 Department of Medicine, Division of Clinical Pharmacology, University of Cape Town, Cape Town, South Africa; 4 Department of Medicine, GF Jooste Hospital, Duinefontein Road, Mannenberg, 7764, Cape Town, South Africa

## Abstract

**Background:**

High rates of mortality and morbidity have been described in sub-Saharan African patients within the first few months of starting highly active antiretroviral therapy (HAART). There is limited data on the causes of early morbidity on HAART and the associated resource utilization.

**Methods:**

A cross-sectional study was conducted of medical admissions at a secondary-level hospital in Cape Town, South Africa. Patients on HAART were identified from a register and HIV-infected patients not on HAART were matched by gender, month of admission, and age group to correspond with the first admission of each case. Primary reasons for admission were determined by chart review. Direct health care costs were determined from the provider's perspective.

**Results:**

There were 53 in the HAART group with 70 admissions and 53 in the no-HAART group with 60 admissions. The median duration of HAART was 1 month (interquartile range 1-3 months). Median baseline CD4 count in the HAART group was 57 × 10^6 ^cells/L (IQR 15-115). The primary reasons for admission in the HAART group were more likely to be due to adverse drug reactions and less likely to be due to AIDS events than the no-HAART group (34% versus 7%; p < 0.001 and 39% versus 63%; p = 0.005 respectively). Immune reconstitution inflammatory syndrome was the primary reason for admission in 10% of the HAART group. Lengths of hospital stay per admission and inpatient survival were not significantly different between the two groups. Five of the 15 deaths in the HAART group were due to IRIS or adverse drug reactions. Median costs per admission of diagnostic and therapeutic services (laboratory investigations, radiology, intravenous fluids and blood, and non-ART medications) were higher in the HAART group compared with the no-HAART group (US$190 versus US$111; p = 0.001), but the more expensive non-curative costs (overhead, capital, and clinical staff) were not significantly different (US$1199 versus US$1128; p = 0.525).

**Conclusions:**

Causes of early morbidity are different and more complex in HIV-infected patients on HAART. This results in greater resource utilization of diagnostic and therapeutic services.

## Background

Highly active antiretroviral therapy (HAART) has dramatically improved the prognosis of HIV infection in high-income countries by decreasing mortality, AIDS illnesses, and hospitalization [1-3]. In low-income countries patients start HAART with lower baseline CD4 counts than in high-income countries [4]. Severely immune suppressed patients remain at increased risk of several complications within the first few months of starting HAART. First, although HAART reduces the incidence of AIDS-defining events, the incidence rates remain high in the first few months [5]. Second, immune reconstitution inflammatory syndrome (IRIS) reactions occur more commonly with lower baseline CD4 counts [6]. Third, many adverse drug reactions to antiretroviral agents occur more commonly with advanced disease [7]. Therefore it is not surprising that there are high rates of early morbidity [8-10] and mortality [4,8] in patients starting HAART in low-income countries.

Few studies have assessed the causes of early morbidity on HAART in sub-Saharan African ART programmes. Most studies report only HIV-related morbidity [8,9] or on specific diseases like tuberculosis [11]. The cohorts in the two West African studies [8,9] had considerably higher baseline CD4 counts (71% >200 × 10^6 ^cells/L and median 290 × 10^6 ^cells/L) than is typical for sub-Saharan African programmes. A South African study included patients more typical of the region, but did not include IRIS as a diagnostic category [10]. IRIS was also not reported in the West African studies [8,9].

We conducted a cross-sectional study to determine whether the reasons for hospitalization (including all causes), outcomes, and resource utilization during hospital admissions were different in HIV-infected patients on HAART compared with HIV-infected patients not on HAART in South Africa, a country with a very high HIV prevalence.

## Methods

### Study Site Description

The study was conducted at G.F. Jooste Hospital, a secondary-level hospital providing care for indigent patients referred from primary health-care providers and clinics in the poor urban communities of metropolitan Cape Town.

A cross-sectional study was conducted to assess the spectrum of disease and resource utilization of medical admissions to G.F. Jooste Hospital among HIV-infected patients on HAART (HAART group) compared with those not on HAART (no-HAART group) during a nine-month period from July 2003 through March 2004. The patients on HAART accessed antiretroviral therapy predominantly from donor-funded programs in G.F. Jooste Hospital's catchment area, because the national public sector HAART roll-out program only commenced in 2004. The donor-funded programs included Médecins sans Frontières (MSF) at Khayelitsha [12]; Hannan Crusaid at Gugulethu [13] (both operated at public sector primary care clinics); and the joint Nelson Mandela Foundation-South African Medical Association's Tshepang project, GF Jooste Hospital clinic. These programs followed the 2002 WHO guidelines for HAART in resource-limited settings, initiating HAART at CD4+ counts < 200 × 10^6 ^cells/L or with clinical AIDS [14].

Patients in the HAART group were identified from a registry of HIV-infected patients on HAART who were admitted to the medical wards. The registry was maintained by two resident infectious disease physicians. Patients in the no-HAART group were matched by gender, month of admission, and age group (18-29; 30-49; and >49 years) to correspond with the first admission of each patient in the HAART group. Subsequent admissions of patients in both groups during the study period were also captured.

The study was approved by the University of Cape Town's Research Ethics Committee.

### Data capture and analysis

The following data were extracted by retrospective chart review: demographic information, WHO clinical stage prior to admission, tuberculosis treatment or prophylaxis, cotrimoxazole prophylaxis, HAART regimen and date of commencement, treatment administered in hospital (medication, fluids, or blood products), investigations during hospitalization (cross-checked with the laboratory's electronic database), CD4+ cell count within six months of admission (if this was unavailable, the total lymphocyte count was recorded), length of stay, discharge diagnoses, discharge disposition (home/step-down facility/tertiary hospital), and survival. The data were recorded in a spreadsheet (Excel, version 97) and checked for illogical or missing entries by the database analyst. Data were analyzed with EpiInfo, version 6. Barlett's test was used for homogeneity of variance, which was shown to differ between the two groups, therefore the Kruskal-Wallis test was used to analyse differences between the outcomes and costs of the two groups.

The study physician classified the primary reason for admission (identified from the discharge diagnoses and a chart review) into the following diagnostic categories: AIDS-defining events, adverse drug reactions, HIV-related conditions, and other medical conditions not necessarily related to HIV. In the WHO clinical staging classification, pulmonary tuberculosis is defined as stage 3, whilst extrapulmonary tuberculosis is stage 4 (AIDS). Because it was not always clear from discharge diagnoses which form of tuberculosis patients had, we did not separate tuberculosis by site and included all forms of tuberculosis as AIDS illnesses. HIV-related conditions were recorded primarily according to WHO clinical stages 1 to 4 [15]. IRIS was defined as paradoxical deterioration of an opportunistic disease that was diagnosed prior to HAART commencement.

### Costing

Costing took the perspective of the health-care provider. The median cost per inpatient day was calculated separately for non-curative care costs and for patient-specific diagnostic and therapeutic costs. Patient-specific laboratory and therapeutic costs were calculated using a standard ingredients approach, which involves linking the usage of resources to the cost of each resource [16]. As the study did not use timesheets to calculate the time spent by clinical staff with each patient, costs for these clinical staff were included in non-curative care costs.

Non-curative care costs, including recurrent overhead and hotel costs, capital costs, and clinical staff costs, were calculated by the study economist using a standard step-down approach [17]. Hotel costs include rent on buildings, utilities, ward staff (laundry, kitchen, building maintenance), administration, and other nonspecific services and stores that cannot be directly linked to patient utilization. Hotel costs at G.F. Jooste medical wards and its step-down ward, Carnation, were assumed to be the same. Capital costs include assets that are not used up in one year, such as buildings, furniture, and medical equipment. The replacement costs of buildings and equipment were based on data from the National Health Facilities Audit undertaken by the National Department of Health (Rod Bennet, personal communication). These data were annualized using an estimated working life of 30 years for buildings and 5 years for equipment at a rate of 8 percent. This real interest rate is the return on long-term government bonds in South Africa and is similar to the rate of 10 percent in a comparable secondary facility in East Africa [18].

Clinical staff costs included salaries of doctors (attending physicians, medical registrars, and interns) and nurses (chief nurses, registered nurses, nurses-in-training, and nurse assistants). The clinical staff cost per inpatient day was calculated by establishing the full cost of employment (including benefits) of doctors and nurses together with the number of full time equivalent staff working in the medicine wards. Multiplying these two allowed us to estimate the annual cost of these clinical inputs. The clinical staff cost per inpatient day was then calculated by the formula:

Annual clinical staff cost/(number of beds × average bed occupancy × 365 days per year).

Patient-specific diagnostic and therapeutic costs included laboratory investigations, radiology, intravenous fluids and blood, and non-ART medications. Laboratory costs were provided by the National Health Laboratory Service. Drug costs were obtained from the hospital pharmacy price list, which is derived from the 2003 Provincial Tender Price List. Radiology and special investigations were costed from the central government's Uniform Fee Schedule (UPFS) 2002, inflated to the 2003 level using the consumer price index, less mortgage bonds [19,20].

All costs were converted from South African Rands to U.S. dollars at the average bank rate at the midpoint of the study (November 2003): U.S. $1 = 6.73231 Rand [21]. The total cost for each admission was calculated by the length of stay multiplied by non-curative costs per day, plus total diagnostic and therapeutic costs.

## Results

Between July 2003 and March 2004, 72 admissions of HIV-infected individuals on HAART met the inclusion criteria, but 2 patients' folders were lost, leaving 70 admissions analyzed (53 patients, 17 with 2 admissions). Two-hundred thirty-seven HIV-infected individuals not on HAART who had been admitted to G.F. Jooste during the study period were identified, and 204 patient folders from those admissions were found. Fifty three patients in the no-HAART group were matched with the first admission of the 53 patients in the HAART group representing 60 admissions (6 were admitted twice, and one was admitted thrice). Five patients in the no-HAART group were matched by season of admission as no exact month match could be made.

Ninety-seven percent of patients admitted on HAART were on a non-nucleoside reverse transcriptase inhibitor (NNRTI) regimen with dual NRTI. One patient was on self-funded dual NRTI antiretroviral therapy, but was included in the study in the HAART group. The median duration of antiretroviral therapy was 1 month (interquartile range 1-3 months; range 1-7 months).

The demographic and clinical characteristics of the patients are summarized in Table 1. Prior AIDS was more common in the HAART group admissions (p = 0.034). There was no significant difference in median CD4 counts between the HAART and the no-HAART groups, but only 11 no-HAART admissions had CD4 counts performed. Total lymphocyte count (TLC) was available for 10 additional no-HAART admissions and was ≤1250 × 10^6 ^cells/L in 4 admissions (which corresponds to a CD4 count of < 200 × 10^6 ^cells/L [22]). Availability of viral loads was very limited at the time of the study, owing to the test's high cost. As viral loads were only available in 15 (21%) HAART admissions and none of the no-HAART group, this parameter was not analyzed.

**Table 1:** Demographics and characteristics of HIV-infected patient admissions at G.F. Jooste Hospital.

Variable	HAART	No-HAART	p-value
**Number of Admissions**			
Total admissions	70	60	
First admissions	53	53	
**Median Age**	32 (IQR 29-35)	32 (IQR 27-40)	p = 0.871
**Gender**			p = 0.647
Men	20 (29%)	15 (25%)	
Women	50 (71%)	45 (75%)	
**Referral source**			p = 0.000
public primary-care clinic	52 (74%)	29 (48%)	
secondary hospital clinic	10 (14%)	2 (3%)	
tertiary hospital clinic	3 (4%)	0	
private medical doctor	4 (6%)	16 (27%)	
not recorded	1 (1%)	13 (22%)	
**Prior WHO Stage**			p = 0.040
Stage I	0 (0%)	3 (5%)	
Stage II	0 (0%)	0 (0%)	
Stage III*	13 (19%)	18 (30%)	
Stage IV*	57 (81%)	39 (65%)	
**CD4 Count **× 10^6 ^cells/L			
Number performed	64 (91%)	11 (18%)	
Median CD4 count	57 (IQR 15-115)	16 (IQR 10-316)	p = 0.96
**Cotrimoxazole prophylaxis**	36	7	p = < 0.001

*All forms of tuberculosis were regarded as stage IV as it was not always clear from discharge diagnoses whether tuberculosis was pulmonary or extrapulmonary

Table 2 lists the primary reasons for hospital admission among patients in the HAART and no-HAART groups. AIDS-defining events constituted the main reason for admission in both groups, but was significantly more common in the no-HAART group. Conversely, drug reactions were significantly more common in the HAART group, in whom it was the second-most frequent reason for admission. Drug reactions occurred as diagnoses other than the primary reason for admission in a further 33 and 2 admissions in the HAART and no-HAART groups respectively. Antiretrovirals accounted for 35/57 (61%) of all of the drug reactions in the HAART group, whilst 6/6 (100%) of all the drug reactions in the no-HAART group were from antitubercular drugs. IRIS, included in the AIDS category, was the primary reason for admission in seven (10%) of the HAART admissions and all occurred within one month of the start of ART. IRIS cases were paradoxical deterioration of cryptococcal meningitis (n = 3) and tuberculosis (n = 4). The median number of diagnoses per admission was 6 (IQR 5-7) and 5 (IQR 4-7) in the HAART and no-HAART groups respectively (p = 0.18). Tuberculosis or its sequelae were listed as diagnoses in 110/130 (85%) admissions, (61 in the HAART group and 49 in the no-HAART group; p = 0.388). The proportion of patients on antitubercular therapy commenced prior to admission was 34% in the HAART group and 20% in the no-HAART group, with a trend toward significance (p = 0.07).

**Table 2:** Primary Reason for Admission

Disease category	HAART (n = 70) n (%)	No-HAART (n = 60) n (%)	p-value
**AIDS**	27* (39%)	**38 (63%)**	**p = 0.005**
Tuberculosis	17	28	
Cryptococcal meningitis	5	3	
Cryptosporidiosis	2	0	
Pneumocystis pneumonia	1	6	
Kaposi sarcoma	1	0	
HIV encephalopathy	1	0	
Esophageal candidiasis	0	1	
**Drug reactions**	**24 (34%)**	**4 (7%)**	**p < 0.001**
Hepatitis	7	3	
Stevens-Johnson syndrome	0	1	
Anemia (zidovudine)	6		
Lactic acidosis (stavudine)	5		
Neuropathy (stavudine)	2		
Pancreatitis (stavudine or lamivudine)	1		
Emesis (nevirapine)	1		
Rechallenge HAART	1		
Myopathy (zidovudine)	1		
**HIV-related conditions**	**7 (10%)**	**6 (10%)**	**p = 1**
Severe bacterial infection	6	4	
Chronic diarrhea	1	0	
HIV cardiomyopathy	0	2	
**Other conditions**	**12 (17%)**	**12 (20%)**	**p = 0.676**
Aseptic meningitis	5	2	
TB sequelae	3	0	
Deep venous thrombosis	2	4	
Drug overdose	1	0	
Hematologic disorder	1	1	
Renal failure	0	1	
Stroke	0	1	
Diabetic complications	0	1	
Reactive arthritis	0	1	
Palliative care	0	1	

* Includes 7 admissions for IRIS events (cryptococcal meningitis 3 and tuberculosis 4)

Table 3 includes outcomes and length of stay. Overall, there was no significant difference in length of stay or death observed between the two groups. During the admission period, there were 14 total referrals to the area's tertiary hospital, either for special investigations (n = 9) or admission (n = 5). The majority of the 19 deaths during the first admission were among individuals admitted with an AIDS-defining event (n = 10), of whom 6 were HAART admissions and 4 were no-HAART admissions. Three persons on HAART who were admitted with a drug reaction died, one from lactic acidosis. Three HAART cases and 1 no-HAART admission died from conditions not related to HIV. Two deaths in the HAART group were attributed to IRIS.

**Table 3:** Length of Stay and Outcome

Variable	HAART (n = 70)	No-HAART (n = 60)	p-value
**Median Length of Stay, days (IQR)**			
Overall	8.5 (4-16)	8 (5-13)	p = 0.63
Medical ward	8 (4-15)	7 (4-10)	
Step-down ward	10.5 (4-30.5)	9.5 (5.5-14.5)	
			
**Outcome**			
Total admissions			p = 0.139
alive	55 (79%)	53 (88%)	
dead	15 (21%)	7 (12%)	
First admission			p = 0.076
alive	40 (75%)	47 (89%)	
dead	13 (25%)	6 (11%)	
			
**Discharge**	(n = 56)	(n = 54)	
Home	44 (79%)	37 (69%)	
Step-down ward	7 (13%)	12 (22%)	
Tertiary hospital	4 (7%)	4 (7%)	
TB Hospital	1 (2%)	1 (2%)	

The total cost of hospitalization of all of the admissions, 1657 patient days, was US$257,033. In both groups, non-curative costs constituted the greatest proportion of total hospitalization costs (90% HAART; 89% no-HAART). Within non-curative costs, overheads constituted the largest share of the costs (46% HAART; 47% no-HAART). The costs of hospitalization of both groups are depicted in Table 4. Although admission costs were somewhat higher in the HAART group, the difference was not significant. Similarly, there were no significant differences in costs observed between both groups when controlling for admission to G.F. Jooste alone (p = 0.15) or controlling for admission to the Carnation step-down ward (p = 0.76). However, diagnostic and therapeutic costs were higher in the HAART group.

**Table 4:** Median Cost in US$ of Hospitalization

Variable	HAART	No-HAART	p-value
Non curative costs G.F. Jooste plus step-down ward*	$1199 (IQR $564-2257)	$1128 (IQR $705-1764)	p = 0.595
Diagnostic and therapeutic cost **	$191 (IQR $88-323)	$111 (IQR $62-207)	p = 0.001
Total hospitalization cost per admission	$1409 (IQR $724-$2658)	$1304 (IQR $813-2077)	p = 0.525

* Length of stay multiplied by the sum of daily overhead, capital, and clinical staff costs.

** Sum of laboratory investigations, radiology, intravenous fluids and blood, and non-ART medications

## Discussion

We analysed diagnoses, outcomes and resource utilization in 130 secondary hospital HIV admissions comparing patients who recently started HAART with those not on HAART in a high HIV prevalence area. We found that the primary reasons for admission were more likely to be due to adverse drug reactions and less likely to be due to AIDS events in patients on HAART. IRIS emerged as an important cause of early morbidity, accounting for 10% of the primary causes of admission in the HAART group. IRIS together with adverse drug reactions accounted for a third of all deaths in patients on HAART.

The patients in our study were severely immune suppressed, as assessed by WHO clinical staging and CD4 cell counts, which is typical of patients starting HAART in Africa. We found that tuberculosis was by far the commonest diagnosis in both the HAART and no-HAART admissions. In the West African studies tuberculosis was the second commonest diagnosis [8,9], but it was the commonest in another South African study [10], which reflects the higher TB incidence in South Africa. However, we found that adverse drug reactions were more commonly the primary reason for admission among our HAART group than the other South African study [10]. Adverse drug reactions were not assessed in the West African studies. In a recent study of severe adverse drug reactions in South African medical inpatients, among HIV-infected patients those on HAART were 10 times more likely than those not on HAART to be admitted to hospital with an adverse drug reaction [23], which is in keeping with our finding that adverse drug reactions were far more commonly the primary reasons for admission in the HAART group. IRIS accounted for 10% of our admissions and was not assessed in either of the West African studies or the other South African study.

Hospitalization costs are a major component of costs for HIV care in developed countries, particularly in patients with more advanced disease [24]. The hospital sector is where HIV currently has the greatest impact on the public health budgets in South Africa, where HIV admissions account for 25% of all hospital admissions and consume one eighth of the public-health budget [25]. Costs of delivering HAART are in part offset by savings on hospitalization. A South African study of the cost-effectiveness of HAART demonstrated dramatic reductions in the costs of hospitalization and a shift of care delivery to the outpatient setting for patients accessing HAART [26]. We found that there were significantly more costs for diagnostic and therapeutic interventions of patients on HAART compared with controls. The higher resource utilization for diagnostic and therapeutic services in the HAART group likely reflects the greater complexity of diagnosis in these patients. The similar median total costs among both arms of the cohort arise from the fact that the hospitalization costs are being driven by per diem non-curative costs. Higher per admission costs for patients on HAART compared with those not on HAART were also reported in another South African study [27]. In our study, the recording of comorbidities and inclusion of associated diagnostic and therapeutic costs carries forward the work by Guiness et al in Kenya, which was limited to costs related to the primary reason for admission [18].

Our findings have several implications. First, starting HAART at higher baseline CD4 counts would reduce the incidence of IRIS, many adverse drug reactions, and HIV-related morbidity. This would result in a shift from inpatient to outpatient HIV care, which would free substantial resources for the many other competing health care priorities in Africa. Second, the use of safer antiretroviral drugs (e.g. tenofovir or efavirenz instead of stavudine or nevirapine) will reduce morbidity as adverse drug reactions were the primary reason for admission in 34% of our HAART group. The World Health Organization has recommended this strategy in resource-limited settings, but recognizes that agents with better safety profiles are currently more costly [28]. Third, the large burden of tuberculosis we found, which is typical of other African studies, emphasises that prevention, diagnosis and treatment of tuberculosis should be integrated into antiretroviral programmes. Fourth, differential diagnosis and management in patients presenting with early morbidity on HAART is complex and difficult in resource-limited settings with limited diagnostic facilities. The differentiation of IRIS from new opportunistic disease or drug toxicity is particularly complex. Training manuals for health care workers in Africa should include the causes of early morbidity on HAART, as well as the importance of their early recognition and appropriate management.

Our study has a number of limitations. We collected data retrospectively, which is always less reliable and complete than in prospective studies. We could only detect large difference between patients in the HAART and no-HAART groups given our relatively small sample size and comparisons between the two groups should be interpreted with caution. Our study was also only limited to inpatients and should not be extrapolated to outpatients. However, detailed costing was done on all 130 admissions, which involved a considerable amount of data collection. Limitations of our cost analysis include lack of a detailed assessment of clinical staffing costs, and exclusion of the indirect and direct non-health care costs borne by patients and their families. Importantly, future cost savings from HAART, notably reduction in rates of hospitalisation, were not assessed. Our study was conducted early on in the roll out of antiretroviral therapy when the numbers of patients on HAART in our catchment area was considerably lower than it is currently, but CD4 counts at HAART initiation were lower than they are currently, which should result in lower hospitalization rates and possibly different reasons for hospitalisation. CD4 counts were discouraged on inpatients as a cost-saving measure, but were done as outpatients. Because only a minority of patients in the no-HAART group was in HIV care while all those on HAART were, we had access to CD4 counts in most of the HAART group and few of the no-HAART group. Finally, although access to diagnostic and therapeutic interventions was similar for both groups, we could not exclude the possibility that clinicians looking after patients on HAART might have been more active in investigating and treating them due to a perceived better prognosis.

## Conclusion

In conclusion, our study provides disease and economic information that may assist with the planning and delivery of comprehensive HIV/AIDS care in Africa. A high proportion of admissions for patients on HAART is due to adverse drug reactions or IRIS. Further large prospective cohort studies should be conducted to identify the nature of the early morbidity on HAART in Africa more accurately, and to identify strategies to prevent, diagnose and manage these morbidities.

## Competing interests

The authors declare that they have no competing interests.

## Authors' contributions

GM, KR, and GAM conceived the study. TKS assisted with the study design, collected the data, developed the database, and wrote the paper. SC, the study health economist, calculated the non-curative costs and contributed to writing the costing section. JHS analyzed the database. GM supervised the study. All authors read and approved the final manuscript.

## Pre-publication history

The pre-publication history for this paper can be accessed here:

http://www.biomedcentral.com/1471-2334/9/205/prepub
